# Association between the aggregate index of systemic inflammation and CKD: evidence from NHANES 1999–2018

**DOI:** 10.3389/fmed.2025.1506575

**Published:** 2025-03-10

**Authors:** Dongli Huang, Hang Wu

**Affiliations:** Department of Nephrology, Bishan Hospital of Chongqing Medical University (Bishan Hospital of Chongqing), Chongqing, China

**Keywords:** the aggregate index of systemic inflammation, chronic kidney disease, estimated glomerular filtration rate, cross-sectional study, inflammation markers

## Abstract

**Purpose:**

We aimed to investigate the potential association between the aggregate index of systemic inflammation (AISI) and chronic kidney disease (CKD).

**Patients and methods:**

This study analyzed data from the National Health and Nutrition Examination Survey (NHANES) spanning 1999 to 2018. CKD was defined as either an estimated glomerular filtration rate (eGFR) of less than 60 mL/min/1.73 m^2^ or the presence of albuminuria, defined as a urine albumin-to-creatinine ratio (ACR) of 30 mg/g or higher. Low eGFR is an eGFR of less than 60 mL/min/1.73 m^2^. Multivariate regression analysis, smoothed curve fitting, and subgroup analyses were conducted to investigate the relationship between the Inflammatory status index (AISI) and CKD. The receiver operating characteristic (ROC) curve analysis was used to evaluate its ability to identify CKD and low eGFR. The AISI was transformed using the natural logarithm (Ln) for statistical analysis.

**Results:**

Of the 50,768 recruits, 49.86% were male. The prevalence of CKD and low eGFR was 20.31% and 8.57%, respectively. Ln-AISI was positively associated with CKD (OR = 1.24; 95% CI: 1.19, 1.28) and low eGFR (OR = 1.17; 95% CI:1.11, 1.24). Smooth curve fitting revealed a positive association between AISI and CKD and low eGFR. Subgroup analysis and interaction tests indicated that stratifications did not significantly alter the association between AISI and CKD and low eGFR. Threshold effect analysis indicated that this relationship became more pronounced when Ln-AISI exceeded 5.2 (AISI > 181.27). The ROC analysis showed that AISI had better discrimination and accuracy for identifying CKD and low eGFR compared to other inflammatory indicators [lymphocyte count (LYM), systemic immune-inflammation index (SII), platelet-to-lymphocyte ratio (PLR), and the product of platelet count and neutrophil count (PPN)].

**Conclusion:**

AISI was significantly and positively correlated with the prevalence of CKD, and this relationship was more potent when AISI was greater than 181.27. Compared with other inflammatory indicators, AISI was more effective in identifying CKD.

## Introduction

Chronic kidney disease (CKD), defined by a low estimated glomerular filtration rate (eGFR) or elevated albuminuria, is recognized as one of the top 10 global prognostic factors (1). Patients with CKD commonly exhibit hypertension, atherosclerosis, abnormal lipid metabolism, chronic inflammation, and oxidative stress (2). Chronic inflammation and oxidative stress worsen endothelial damage, forming unstable atherosclerotic plaques (3–5). Inflammation, obesity, diabetes mellitus, hypertension, and hyperlipidemia are established risk factors for CKD (6, 7). Inflammation, as a critical risk factor, is pivotal in developing effective therapeutic strategies to prevent the onset and progression of CKD in clinical practice.

The aggregate index of systemic inflammation (AISI), also known as the pan-immune-inflammation value (PIV), is a marker that evaluates systemic inflammation through key components of the complete blood count (CBC), including neutrophils (NEU), platelets (PLT), monocytes (MONO), and lymphocytes (LYM). AISI is calculated using the formula: AISI = (NEU * PLT * MONO) / LYM. AISI has been investigated as a novel inflammatory marker in patients with rheumatoid arthritis, hypertension, proteinuria, and certain cancers (8–12). It has demonstrated the ability to identify specific diseases and assess prognosis. Previous studies have shown that inflammation is associated with kidney damage and that AISI is a more comprehensive inflammatory indicator than SII and SIRI. It can more comprehensively evaluate the systemic inflammatory state and is superior in assessing proteinuria (10). However, no studies have investigated the relationship between AISI and CKD or low eGFR. Therefore, this study aims to explore the association between AISI and CKD using data from the National Health and Nutrition Examination Survey (NHANES).

## Methodology

### Study population

**FIGURE 1:**
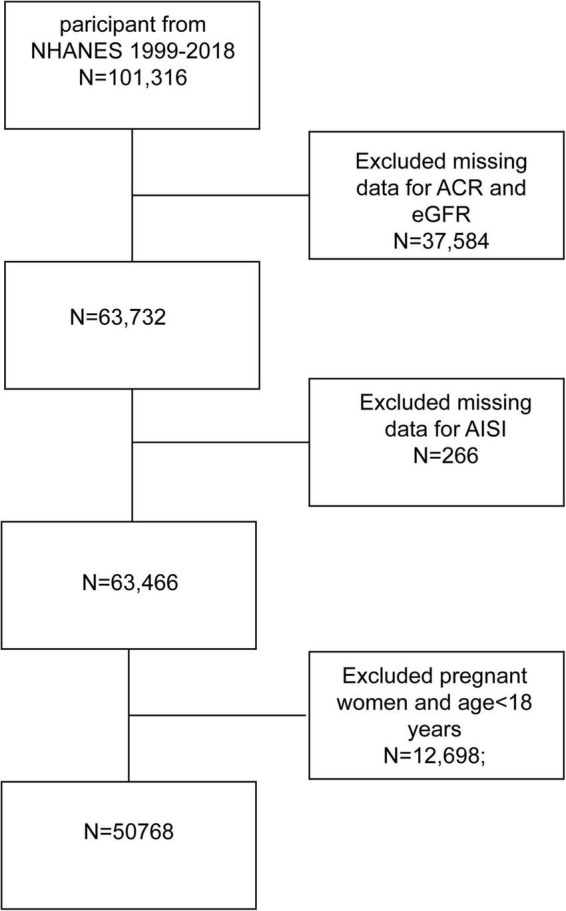
Flowchart of participant selection. NHANES, National Health and Nutrition Examination Survey; AISI, aggregate index of systemic inflammation.

The National Health and Nutrition Examination Survey (NHANES) is a key initiative sponsored by the US Centers for Disease Control and Prevention (CDC) to evaluate the health and nutritional status of the US population. This study utilized data from 10 NHANES cycles spanning 1999 to 2018, comprising 101,316 participants. After excluding participants with missing albumin-creatinine ratio (ACR), eGFR, and AISI data, pregnant women, and those under 18, the final cohort comprised 50,768 subjects. The National Center approved the study for the Health Statistics Research Ethics Review Board, and all participants provided informed consent. Detailed information on the publicly available NHANES study design and data can be accessed at https://wwwn.cdc.gov/nchs/nhanes/default.aspx. The inclusion and exclusion process is illustrated in Figure 1.

### Aggregate index of systemic inflammation definition

AISI is a biomarker to evaluate systemic inflammation. It is derived from the complete blood count (CBC) components. Using a single-beam photometer involves automated sample processing, including dilution, mixing, and hemoglobin determination. In the NHANES Mobile Examination Center (MEC), the Beckman Coulter DxH 800 device conducts CBC analysis on blood samples, providing blood cell distribution, including neutrophils (NEU), platelets (PLT), monocytes (MONO), and lymphocytes (LYM). AISI is calculated as (NEU * PLT * MONO)/LYM (13). AISI was log-transformed (Ln) for the regression analysis due to its right-skewed distribution.

### Diagnosis of CKD

According to prior studies on CKD using the NHANES database, CKD was diagnosed based on proteinuria or an eGFR below 60 mL/min/1.73 m^2^ (14–17). The eGFR was calculated using the chronic kidney disease epidemiology collaboration (CKD-EPI) equation with standardized creatinine (18). An eGFR of less than 60 mL/min/1.73 m^2^ was classified as low eGFR. Previous studies have demonstrated a relationship between AISI and proteinuria (10); thus, our study’s outcome variables were CKD and low eGFR.

### Selection of covariates

Covariates that could potentially influence the association between AISI and proteinuria were included in this study. The covariates included sex (male/female), age (< 60 years/≥ 60 years), ethnicity (Mexican American/Other Hispanic/Non-Hispanic White/Non-Hispanic Black/Other), an education level (less than high school/high school or equivalent/college graduate or above), marital status (married/widowed/divorced or separated/never married/living with a partner), poverty-to-income ratio (PIR: < 1.3, 1.3–3.49, ≥ 3.5), smoking status (current smokers/nonsmokers/former smokers), alcohol consumption (current drinkers/nondrinkers/former drinkers), physical activity (vigorous activity/moderate activity), body mass index (BMI: < 25, 25–30, > 30 kg/m^2^), hypertension (yes/no), diabetes mellitus (yes/no), alanine aminotransferase (ALT, IU/L), aspartate aminotransferase (AST, IU/L), triglycerides (TG, mmol/L), low-density lipoprotein cholesterol (LDL-C, mmol/L), albumin (ALB, g/L), and blood uric acid (UA, mg/dL). Physical activity was categorized as vigorous (yes/no) or moderate (yes/no). Participants who reported smoking at least 100 cigarettes in their lifetime and were smoking at the time of the survey were classified as current smokers. Former smokers were defined as those who had smoked at least 100 cigarettes in their lifetime but were not smoking at the time of the survey. Additionally, men who had smoked fewer than 100 cigarettes in their lifetime were classified as non-smokers. A non-drinker was a participant who answered “no” to having consumed at least 12 alcoholic beverages in their lifetime or any single year. In this study, men who answered “yes” to having consumed 12 alcoholic drinks in their lifetime or any single year but had not consumed any in the past 12 months were classified as former drinkers. Participants who answered “yes” to consuming 12 alcoholic beverages in their lifetime or any single year and had consumed at least one in the past 12 months were classified as current drinkers. Participants were considered diabetic if a physician had diagnosed them, had a fasting blood glucose level ≥ 7.0 mmol/L, glycosylated hemoglobin ≥ 6.5%, a 75 g oral glucose tolerance test result ≥ 200 mg/dL, or were using glucose-lowering medications or insulin. Hypertension was defined as systolic blood pressure ≥ 130 mmHg, diastolic blood pressure ≥ 80 mmHg, a doctor’s diagnosis of high blood pressure, or current use of prescription medication for hypertension. Detailed measurement procedures for these variables are publicly available at https://wwwn.cdc.gov/nchs/nhanes/default.aspx.

### Statistical analysis

In line with the Centers for Disease Control and Prevention (CDC) recommendations, all statistical analyses accounted for the complex sample design of a multistage cohort survey. Continuous variables were expressed as means and standard deviations, and categorical variables were expressed as percentages. Differences in continuous variables across AISI tertiles were assessed using the Kruskal-Wallis rank sum test, while differences in categorical variables across AISI tertiles were assessed using chi-square tests. We found that the AISI data is skewed. Therefore, we must perform an Ln transformation on the values before performing the statistical analysis (Supplementary Figures 1, 2). We used multivariate regression models to examine the association between Ln-AISI and CKD and low eGFR (Models 1–3). Model 1 was unadjusted for any covariates. Model 2 adjusted for sex, age, and race. Model 3 adjusted for gender, age, race, albumin (Alb), body mass index (BMI), education level, marital status, poverty income ratio (PIR), serum uric acid (UA), triglycerides (TG), low-density lipoprotein (LDL), diabetes, alcohol consumption, hypertension, vigorous and moderate physical activity, smoking status, alanine aminotransferase (ALT), and aspartate aminotransferase (AST). To evaluate robustness, we transformed AISI from a continuous variable to a categorical variable (tertiles) for sensitivity analysis. We addressed nonlinearity by applying smooth curve fitting and generalized additive models (GAM). When nonlinear correlations were present, a two-stage linear regression model (segmented regression) was fitted to each interval to estimate threshold effects. A two-step recursive method was employed to further identify breakpoints (K) (The specific methods and explanations of the principles can be found in Supplementary Data Sheet 1). Subgroup analyses were conducted using stratified multivariate logistic regression models [sex, age, poverty-to-income ratio (PIR), BMI, hypertension, diabetes, smoking, alcohol consumption, education, race, and physical activity] to assess the association between AISI and CKD and low eGFR. These stratification variables were also considered as potential effect modifiers to evaluate the heterogeneity of the associations between subgroups. Finally, we compared the ability of AISI and other inflammatory markers (LYM, SII, PLR, PPN) to identify CKD and low eGFR using receiver operating characteristic (ROC) curves and area under the curve (AUC) values. Multiple imputations were applied to handle missing values in categorical and continuous variables. All statistical analyses were performed using R version 4.1.3 and Empower software.^1^ Statistical significance was defined as a *p*-value < 0.05.

## Results

### Baseline characteristics of participants

The study included 50,768 participants, of which 25,311 were men (49.86%) and 25,475 were women (50.14%). Of the participants, 16,420 (32.34%) were aged over 60 years, and 10,312 (20.31%) and 4,350 (8.57%) had been diagnosed with CKD and low eGFR, respectively. Table 1 presents the clinical characteristics of participants stratified by AISI tertiles. Significant differences were found for age, race, education level, marital status, body mass index (BMI), poverty-income ratio (PIR), hypertension, diabetes, alcohol consumption, smoking status, and inflammatory indicators (AISI, LYM, SII, PLR, PPN) (all *p* < 0.001). No significant differences were found for gender and moderate activity (*p* > 0.05).

**TABLE 1:** Baseline characteristics of NHANES participants, 1999–2018.

Variables	Tertiles of Ln-AISI			*P*-value
	**T1**	**T2**	**T3**	
*N*	16,921	16,924	16,923	
ALB, (g/l)	42.73 ± 3.36	42.76 ± 3.32	42.32 ± 3.66	< 0.001
UA, (mg/dL)	5.32 ± 1.40	5.44 ± 1.41	5.58 ± 1.50	< 0.001
AISI	128.28 ± 40.13	254.01 ± 40.00	570.12 ± 377.78	< 0.001
ACR (mg/g)	32.18 ± 290.93	41.93 ± 326.06	64.59 ± 449.94	< 0.001
TG, (mmol/L)	1.42 ± 1.76	1.59 ± 1.83	1.59 ± 1.83	< 0.001
LDL-C (mmol/L)	2.93 ± 0.70	2.94 ± 0.65	2.92 ± 0.62	0.007
ALT, (U/L)	24.85 ± 17.84	25.35 ± 20.31	25.28 ± 31.98	< 0.001
AST, (U/L)	25.11 ± 18.80	25.02 ± 20.69	26.69 ± 18.92	< 0.001
LYM, (1,000 cells/ul)	2.28 ± 3.35	2.16 ± 0.71	2.07 ± 0.73	< 0.001
SII	308.11 ± 114.15	494.05 ± 148.75	848.46 ± 485.75	< 0.001
PLR	108.00 ± 37.76	125.93 ± 43.05	153.70 ± 62.33	< 0.001
PPN	649.66 ± 256.12	1,022.72 ± 327.49	1,636.16 ± 938.36	< 0.001
**eGFR (mL/min/1.73m^2^)**				< 0.001
< 60	1,048 (6.19%)	1,329 (7.85%)	1,973 (11.66%)	
≥ 60	15,873 (93.81%)	15,595 (92.15%)	14,950 (88.34%)	
**BMI, *n* (%)**				< 0.001
< 25	5,926 (35.02%)	5,028 (29.71%)	4,790 (28.30%)	
≥ 25, < 30	5,745 (33.95%)	5,924 (35.00%)	5,632 (33.28%)	
≥ 30	5,250 (31.03%)	5,972 (35.29%)	6,501 (38.42%)	
**Gender, *n* (%)**				0.122
Male	8,358 (49.39%)	8,411 (49.70%)	8,542 (50.48%)	
Female	8,563 (50.61%)	8,513 (50.30%)	4,969 (36.13%)	
**Age, *n* (%)**				< 0.001
< 60	11,889 (70.26%)	11,571 (68.37%)	10,888 (64.34%)	
≥ 60	5,032 (29.74%)	5,353 (31.63%)	6,035 (35.66%)	
**Race, *n* (%)**				< 0.001
Mexican American	2,856 (16.88%)	2,410 (17.55%)	2,922 (21.24%)	
Other Hispanic	1,360 (8.04%)	1,494 (8.83%)	1,321 (7.81%)	
Non-Hispanic White	5,461 (32.27%)	7,687 (45.42%)	8,910 (52.65%)	
Non-Hispanic Black	5,307 (31.36%)	2,938 (17.36%)	2,276 (13.45%)	
Other Race	1,937 (11.45%)	1,490 (8.80%)	1,227 (7.25%)	
**Education, *n* (%)**				< 0.001
Under high school	4,224 (24.96%)	4,246 (25.09%)	4,285 (25.32%)	
High school or equivalent	3,366 (19.89%)	3,598 (21.26%)	3,959 (23.39%)	
College graduate or above	9,331 (55.14%)	9,080 (53.65%)	8,679 (51.29%)	
**Marital status, *n* (%)**				< 0.001
Married	9,119 (53.89%)	9,166 (54.16%)	8,580 (50.70%)	
Widowed	1,167 (6.90%)	1,276 (7.54%)	935 (6.80%)	
Divorced	1,500 (10.92%)	1,452 (10.57%)	1,628 (9.62%)	
Separated	1,554 (9.18%)	1,620 (9.57%)	1,723 (10.18%)	
Never married	3,321 (19.63%)	3,200 (18.91%)	3,322 (19.63%)	
Living with partner	1,248 (7.38%)	1,149 (6.79%)	1,117 (6.60%)	
**PIR, *n* (%)**				< 0.001
< 1.3	4,848 (28.65%)	4,778 (28.23%)	5,183 (30.63%)	
≥ 1.3, < 3.5	7,218 (42.66%)	7,319 (43.25%)	7,385 (43.64%)	
≥ 3.5	4,855 (28.69%)	4,827 (28.52%)	4,355 (25.73%)	
**Diabetes, *n* (%)**				< 0.001
Yes	2,411 (14.25%)	2,545 (15.04%)	2,981 (17.62%)	
No	14,510 (85.75%)	14,379 (84.96%)	13,942 (82.38%)	
**Drink, *n* (%)**				< 0.001
Current drinkers	11,905 (70.36%)	12,038 (71.13%)	11,785 (69.64%)	
Nondrinkers	2,351 (13.89%)	2,171 (23.83%)	1,989 (11.75%)	
Former drinkers	2,665 (15.75%)	2,715 (16.04%)	3,149 (18.61%)	
**Hypertension, *n* (%)**				< 0.001
Yes	8,372 (49.48%)	8,778 (51.87%)	9,615 (56.82%)	
No	8,549 (50.52%)	8,146 (48.13%)	7,308 (43.18%)	
**Vigorous activity, *n* (%)**				< 0.001
Yes	6,471 (38.24%)	5,979 (35.33%)	5,313 (31.40%)	
No	10,450 (61.76%)	10,945 (64.67%)	11,610 (68.60%)	
**Moderate activity, *n* (%)**				0.069
Yes	6,952 (41.09%)	7,163 (42.32%)	7,057 (41.70%)	
No	9,969 (58.91%)	9,761 (57.68%)	9,866 (58.30%)	
**Smoke, *n* (%)**				< 0.001
Current smokers	2,778 (16.42%)	3,201 (18.91%)	4,049 (23.93%)	
Nonsmokers	10,481 (61.94%)	9,747 (51.59%)	8,693 (51.37%)	
Former smokers	3,662 (21.46%)	3,976 (23.49%)	4,181 (24.71%)	
**CKD, *n* (%)**				< 0.001
Yes	2,850 (16.84%)	3,267 (19.30%)	4,195 (24.79%)	
No	14,071 (83.16%)	13,657 (80.70%)	12,728 (75.21%)	

Categorized according to AISI tertiles. AISI, aggregate index of systemic inflammation; BMI, body mass index; CKD, chronic kidney disease; LYM, lymphocyte count; SII, systemic immune-inflammation index; PLR, platelet-to-lymphocyte ratio; PPN, the product of platelet count and neutrophil count.

### The association between AISI and CKD, as well as low eGFR

Table 2 presents the multivariate logistic regression analysis results, indicating a significant association between higher AISI levels and increased prevalence of CKD and low eGFR. This association remained substantial across various models. In Model 1, each one-unit increase in Ln-AISI was significantly associated with a 39% increase in CKD prevalence (OR: 1.39; 95% CI: 1.35–1.44, *p* < 0.001) and a 58% increase in low eGFR prevalence (OR: 1.58; 95% CI: 1.51–1.65, *p* < 0.001). After adjusting for sex, age, and race in Model 2, the association remained significant (CKD OR: 1.37; 95% CI: 1.33–1.42, *p* < 0.001; low eGFR OR: 1.43; 95% CI: 1.36–1.50, *p* < 0.001). Model 3 further adjusted for Alb, BMI, education, marital status, PIR, UA, TG, LDL-C, diabetes, drinking, hypertension, vigorous activity, moderate activity, smoking, ALT, AST, and the association between AISI and increased CKD and low eGFR prevalence remained significant. AISI was also analyzed as a categorical variable (tertiles) to assess sensitivity. Compared to the lowest tertile, the highest tertile was associated with a 63% increase in Model 1 (OR: 1.63; 95% CI: 1.51–1.72, *p* < 0.001). This increase remained consistent in Model 3 (OR: 1.36; 95% CI: 1.28–1.45, *p* < 0.001). Similarly, the prevalence of low eGFR doubled in the crude model (OR: 2.0; 95% CI: 1.85–2.16, *p* < 0.001) and increased by 33% in Model 3 (OR: 1.33; 95% CI: 1.21–1.46, *p* < 0.001) when comparing the highest to the lowest tertile. These findings indicate that in the population, the higher the AISI value, the higher the prevalence of CKD.

**TABLE 2:** Multivariate regression analysis of Ln-AISI with CKD and low eGFR.

	Model 1 OR 95% CI	Model 2 OR 95% CI	Model 3 OR 95% CI
Ln-AISI VS CKD	1.39 (1.35, 1.44)	1.37 (1.33, 1.42)	1.24 (1.19, 1.28)
**Stratified by Ln-AISI tertiles**			
T1	Ref	Ref	Ref
T2	1.18 (1.12, 1.25)	1.20 (1.13, 1.27)	1.12 (1.06, 1.20)
T3	1.63 (1.51, 1.72)	1.60 (1.51, 1.70)	1.36 (1.28, 1.45)
*P* for trend	< 0.001	< 0.001	< 0.001
Ln-AISI VS low eGFR	1.58 (1.51, 1.65)	1.43 (1.36, 1.50)	1.17 (1.11, 1.24)
**Stratified by Ln-AISI quartiles**			
T1	Ref	Ref	Ref
T2	1.29 (1.19, 1.40)	1.23 (1.12, 1.35)	1.12 (1.01, 1.23)
T3	2.00 (1.85, 2.16)	1.75 (1.60, 1.91)	1.33 (1.21, 1.46)
*P* for trend	< 0.001	< 0.001	< 0.001

OR, odds ratio; 95% CI, 95% confidence interval. Model 1: no covariates were adjusted. Model 2: adjusted for gender, age, and race. Model 3: gender, age, race, Alb, BMI, education, marital status, PIR, UA, TG, LDL, diabetes, drink, hypertension, vigorous activity, moderate activity, smoke, ALT, AST.

### Non-linear relationship between AISI and CKD, as well as low eGFR

**FIGURE 2:**
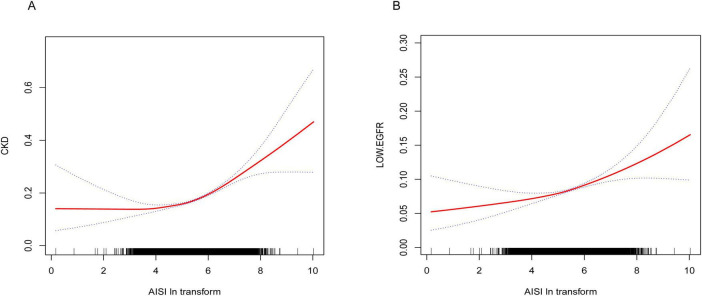
**(A)** Smooth curve fitting diagram of Ln-AISI and CKD in full adjusted models. **(B)** Smooth curve fitting graph of Ln-AISI and low eGFR in full adjusted models.

Results from the smooth curve fitting analysis revealed a positive correlation between Ln-AISI and the prevalence of CKD and low eGFR in the fully adjusted model (see Figure 2). In CKD, this relationship demonstrated a threshold effect characterized by an inflection point at an Ln-AISI value of 5.20, corresponding to an AISI value of 181.27. Below this threshold, the risk of CKD remained relatively stable (OR: 1.08; 95% CI: 0.98–1.19, *p* = 0.1386). However, when the Ln-AISI value exceeds the cutoff point, the risk of CKD increases sharply (OR: 1.38; 95% CI: 1.31–1.46, *p* < 0.001), exhibiting a J-shaped nonlinear relationship (Table 3). There is also a breakpoint at Ln-AISI = 5.12 (AISI = 167.46) for low eGFR, but the effect on both sides of the breakpoint was not statistically significant (*p* = 0.06) (Table 4).

**TABLE 3:** Threshold effects of AISI on CKD analyzed using linear regression models.

	Adjusted OR (95% CI), *P*-value
Fitting by the standard linear model	1.29 (1.24, 1.34) < 0.0001
**Fitting by the two-piecewise linear model**	
Ln-AISI	
Inflection point	5.2
Ln-AISI < 5.2	1.08 (0.98, 1.19) 0.1386
Ln-AISI > 5.2	1.38 (1.31, 1.46) < 0.0001
Log likelihood ratio	< 0.001

Adjusted for gender, age, race, Alb, BMI, education, marital status, PIR, UA, TG, LDL, diabetes, drink, hypertension, vigorous activity, moderate activity, smoke, ALT, and AST. OR, odd ratio; 95% CI, 95% confidence interval.

**TABLE 4:** Threshold effects of AISI on low eGFR analyzed using linear regression models.

	Adjusted OR (95% CI), *P*-value
Fitting by the standard linear model	1.16 (1.10, 1.23) < 0.0001
**Fitting by the two-piecewise linear model**	
**Ln-AISI**	
Inflection point	5.12
Ln-AISI < 5.2	1.00 (0.85, 1.17) 0.9510
Ln-AISI > 5.2	1.22 (1.13, 1.31) < 0.0001
Log likelihood ratio	0.060

Adjusted for gender, age, race, Alb, BMI, education, marital status, PIR, UA, TG, LDL, diabetes, drink, hypertension, vigorous activity, moderate activity, smoke, ALT, and AST. OR, odd ratio; 95% CI, 95% confidence interval.

### Interaction test and subgroup analysis

Subgroup analysis (Table 5) revealed that the association between AISI levels and CKD prevalence was consistent across subgroups, with no significant interaction between groups (*p* for interaction > 0.05). This relationship remained consistent across groups in the subgroup analysis of AISI and low eGFR (Table 6).

**TABLE 5:** Subgroup analysis for the association between AISI and CKD.

Character	OR 95% CI	*P*-value	*P* for interaction
**BMI**			0.9456
Blow 25	1.24 (1.17, 1.33)	< 0.0001	
25–29.9	1.23 (1.15, 1.31)	< 0.0001	
≥ 30	1.25 (1.17, 1.32)	< 0.0001	
**PIR**			0.1592
Blow 1.3	1.18 (1.10, 1.25)	< 0.0001	
1.3–3.5	1.27 (1.20, 1.34)	< 0.0001	
Over 3.5	1.26 (1.17, 1.35)	< 0.0001	
**Drink**			0.1259
Current drinkers	1.22 (1.17, 1.28)	< 0.0001	
Nondrinkers	1.17 (1.07, 1.29)	0.0008	
Former drinkers	1.31 (1.21, 1.41)	< 0.0001	
**Hypertension**			0.2796
Yes	1.25 (1.20, 1.31)	< 0.0001	
No	1.20 (1.12, 1.28)	< 0.0001	
**Diabetes**			0.0600
Yes	1.35 (1.26, 1.45)	< 0.0001	
No	1.26 (1.20, 1.33)	< 0.0001	
**Vigorous activity**			0.1172
Yes	1.17 (1.09, 1.26)	< 0.0001	
No	1.25 (1.20, 1.31)	< 0.0001	
**Moderate activity**			0.8943
Yes	1.23 (1.16, 1.31)	< 0.0001	
No	1.24 (1.18, 1.30)	< 0.0001	
**Gender**			0.7541
Male	1.24 (1.18, 1.31)	< 0.0001	
Female	1.23 (1.17, 1.29)	< 0.0001	
**Smoke**			0.7793
Current smokers	1.21 (1.11, 1.35)	< 0.0001	
Nonsmokers	1.24 (1.18, 1.30)	< 0.0001	
Former smokers	1.25 (1.17, 1.34)	< 0.0001	
**Age**			0.4520
< 60	1.26 (1.17, 1.30)	< 0.0001	
≥ 60	1.29 (1.22, 1.39)	< 0.0001	
**Education**			0.0950
Under high school	1.31 (1.23, 1.40)	< 0.0001	
High school or equivalent	1.21 (1.12, 1.30)	< 0.0001	
College graduate or above	1.20 (1.14, 1.27)	< 0.0001	
**Race**			0.7641
Mexican American	1.29 (1.17, 1.42)	< 0.0001	
Other Hispanic	1.27 (1.11, 1.45)	0.0006	
Non-Hispanic White	1.24 (1.17, 1.31)	< 0.0001	
Non-Hispanic Black	1.21 (1.12, 1.30)	< 0.0001	
Other Race	1.18 (1.04, 1.33)	0.0085	

**TABLE 6:** Subgroup analysis for the association between AISI and low eGFR.

Character	OR 95% CI	*P*-value	*P* for interaction
**BMI**			0.5602
Blow 25	1.23 (1.11, 1.36)	< 0.0001	
25–29.9	1.17 (1.07, 1.28)	0.0007	
≥ 30	1.14 (1.04, 1.24)	0.0058	
**PIR**			0.1911
Blow 1.3	1.09 (0.98, 1.20)	0.1020	
1.3–3.5	1.22 (1.13, 1.32)	< 0.0001	
Over 3.5	1.18 (1.06, 1.32)	0.0032	
**Drink**			0.3632
Current drinkers	1.16 (1.08, 1.25)	< 0.0001	
Nondrinkers	1.25 (1.10, 1.42)	< 0.0001	
Former drinkers	1.15 (1.04, 1.26)	0.0058	
**Hypertension**			0.1921
Yes	1.15 (1.08, 1.22)	< 0.0001	
No	1.26 (1.11, 1.43)	0.0004	
**Diabetes**			0.6602
Yes	1.19 (1.08, 1.31)	0.0002	
No	1.16 (1.09, 1.24)	< 0.0001	
**Vigorous activity**			0.5605
Yes	1.13 (0.98, 1.30)	0.0889	
No	1.18 (1.11, 1.25)	< 0.0001	
**Moderate activity**			0.9565
Yes	1.18 (1.07, 1.49)	0.0007	
No	1.17 (1.09, 1.25)	< 0.0001	
**Gender**			0.1984
Male	1.13 (1.05, 1.22)	0.0014	
Female	1.21 (1.12, 1.31)	< 0.0001	
**Smoke**			0.1855
Current smokers	1.18 (1.01, 1.38)	0.0399	
Nonsmokers	1.23 (1.13, 1.33)	< 0.0001	
Former smokers	1.11 (1.02, 1.20)	0.0202	
**Age**			0.2789
< 60	1.26 (1.10, 1.46)	0.0013	
≥ 60	1.16 (1.09, 1.23)	< 0.0001	
**Education**			0.6720
Under high school	1.20 (1.19, 1.32)	0.0002	
High school or equivalent	1.13 (1.01, 1.26)	0.0333	
College graduate or above	1.18 (1.08, 1.27)	< 0.0001	
**Race**			0.1790
Mexican American	1.23 (1.03, 1.48)	0.0219	
Other Hispanic	1.14 (0.90, 1.45)	0.2881	
Non-Hispanic White	1.12 (1.04, 1.20)	0.0028	
Non-Hispanic Black	1.18 (1.05, 1.32)	0.0067	
Other Race	1.49 (1.18, 1.88)	0.0007	

### ROC analysis

**FIGURE 3:**
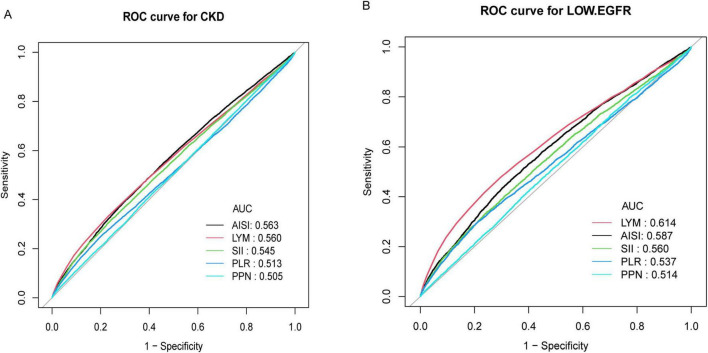
**(A)** ROC curve and AUC value of five inflammatory indicators (LYM, SII, PLR, PPN, and AISI) for identifying CKD; **(B)** ROC curve and AUC value of five inflammatory indicators (LYM, SII, PLR, PPN, and AISI) for identifying low eGFR.

The AUC was calculated to compare the performance of AISI with other inflammatory markers (LYM, SII, PLR, PPN) in identifying CKD and low eGFR (Figure 3). For identifying CKD, the AUC of AISI was 0.5629 (95% CI: 0.5566–0.5692), which was higher than that of the other four inflammatory markers. Additionally, there were statistically significant differences between the AUCs of AISI and those of SII, PLR, and PPN (all *P* < 0.05), but no significant difference between the AUC of AISI and that of LYM (AUC = 0.5605, 95% CI: 0.5540–0.5669, *p* = 0.5641). However, the AUC of AISI was greater than that of LYM. Furthermore, LYM was the best marker for identifying low eGFR (AUC = 0.6142, 95% CI: 0.6048–0.6232) (Table 7).

**TABLE 7:** Comparison of AUC values between AISI and other inflammatory markers in identifying CKD and low eGFR.

Test	AUC	95% CI low	95% CI upp	Best threshold	Specificity	Sensitivity	*P* for different in AUC
**CKD**							
AISI	0.5629	0.5566	0.5692	337.10	0.6955	0.3989	reference
LYM	0.5605	0.5540	0.5669	1.65	0.7705	0.3304	0.5641
PLR	0.5126	0.5061	0.5191	160.69	0.8064	0.2437	< 0.0001
PPN	0.5055	0.4992	0.5117	1080.90	0.5895	0.4228	< 0.0001
SII	0.5452	0.5387	0.5516	660.43	0.7582	0.3157	< 0.0001
**Low eGFR**							
LYM	0.6142	0.6048	0.6236	1.75	0.7033	0.4779	reference
AISI	0.5870	0.5779	0.5960	299.15	0.6193	0.5140	< 0.0001
PLR	0.5367	0.5271	0.5463	159.07	0.7960	0.2906	< 0.0001
PPN	0.5138	0.5049	0.5228	1274.65	0.2950	0.7031	< 0.0001
SII	0.5604	0.5511	0.5697	663.38	0.7538	0.3391	< 0.0001

AUC, area under the curve; 95% CI, 95% confidence interval.

### Sensitivity analysis

This is a cross-sectional study and we show how the sample size was calculated (Supplementary Data Sheet 1). Inflammation markers were important covariates in this study. Due to limitations of the NHANES database, CRP data were available only from 1999 to 2010. After applying our inclusion and exclusion criteria, 25,418 participants (50.07%) in the final dataset had CRP data. However, there was a large amount of missing data, and direct inclusion in the analysis could introduce significant bias. Therefore, we excluded it from the covariate adjustment in our study. We performed a sensitivity analysis using multiple imputations to address all missing values, including CRP and included it in Model 3 for logistic regression (Supplementary Table 1). The results of this analysis were consistent with those of the current study. After including CRP as a covariate in Model 3, the ORs for Ln-AISI and CKD were 1.21 (95% CI: 1.17–1.26), and for Ln-AISI and low eGFR, the ORs were 1.21 (95% CI: 1.14–1.28), respectively. Additionally, we conducted a sensitivity analysis without CRP interpolation, using only participants with available CRP data for multivariate logistic regression (see Supplementary Table 2). The results remained consistent.

Furthermore, our study excluded participants with missing ACR, eGFR, and AISI data. Although this direct exclusion method is commonly employed, it may still introduce bias. To address this, we performed a sensitivity analysis in which all participants were retained, and multiple imputation was applied to the entire population of 101,316 without exclusion criteria. Regression analysis was then performed using the three models of this study (see Supplementary Table 3). Under model 3, which adjusted for all covariates, the OR value of Ln (AISI) and CKD were 1.14 (95% CI: 1.11–1.18) for CKD and 1.28 (95% CI: 1.11–1.25) for low eGFR. The direction of effect was consistent with our findings, and the results were significant.

## Discussion

In this cross-sectional study of 50,768 adults, multivariate logistic regression analysis identified a positive correlation between AISI and CKD and low eGFR. The threshold effect of the non-linear relationship between AISI and CKD was identified through smooth curve fitting. A J-shaped association between AISI and CKD was found, with a breakpoint at 181.27. Additionally, a positive correlation was also observed between AISI and low eGFR. Subgroup analysis and interaction tests revealed no significant differences in the association between AISI and CKD across different populations. ROC analysis showed that AISI was the best indicator for identifying CKD compared with other inflammatory indicators (LYM, SII, PLR, PPN).

Previous studies on inflammatory markers and chronic kidney disease (CKD) have shown that the systemic immune-inflammation index (SII) and systemic inflammation response index (SIRI) are significantly associated with CKD prevalence and mortality in the US population (19, 20). Inflammation plays a crucial role in the development and progression of kidney disease. The aggregate index of systemic inflammation (AISI), a novel inflammatory marker introduced in 2018 (21), has since been studied for its clinical significance in various diseases, including idiopathic pulmonary fibrosis, COVID-19, non-small cell lung cancer, acute coronary syndrome, and multiple types of cancer (11, 12, 22–24). To our knowledge, this is the first study to investigate the association between AISI and CKD prevalence. A previous study of 369 peritoneal dialysis patients, with a median follow-up of 32.83 months, found that those with higher AISI levels had lower survival rates (25). Another cross-sectional study on AISI and proteinuria indicated that when log2AISI exceeds 7.25, the risk of albuminuria significantly increases, similar to our findings (10). Similarly, elevated AISI levels have been associated with an increased risk of cardiovascular mortality in hypertensive patients (9, 26), and kidney damage is closely linked to endothelial injury in the renal tubules caused by hypertension (27).

Chronic systemic inflammation is key to developing and progressing CKD (28). Elevated AISI levels may promote the development of CKD and the decline of eGFR through these mechanisms. Cytokines and chemokines released during inflammation (e.g., IL-6 and TNF-α) directly damage glomerular and tubular cells and promote mesangial cell proliferation and matrix deposition, ultimately leading to glomerulosclerosis (29). Specifically, elevated IL-6 and TNF-α levels are closely linked to renal inflammation. These factors activate downstream signaling pathways (e.g., NF-κB), promoting the infiltration and activation of inflammatory cells, which exacerbates renal damage. Systemic inflammation also induces oxidative stress, leading to the overproduction of reactive oxygen species (ROS). These free radicals directly damage renal cell DNA, proteins, and lipids and exacerbate kidney function through apoptosis and necrosis (30). Studies have shown that excessive ROS production is closely associated with dysfunction of tubular cells. Additionally, inflammation impairs endothelial function, affecting renal blood perfusion and filtration, thereby promoting proteinuria (31). Endothelial dysfunction-induced vasoconstriction and increased permeability worsen renal ischemia, further amplifying the inflammatory response. Persistent inflammation also activates pro-fibrotic factors, such as transforming growth factor beta (TGF-β), which promotes renal interstitial fibrosis and reduces kidney filtration capacity (32). TGF-β induces structural remodeling and kidney dysfunction by activating fibroblasts and promoting collagen deposition.

This study has several key strengths. This is the first study to examine the relationship between AISI and CKD using the NHANES database, offering a novel perspective on the link between inflammation and CKD through AISI. These data are drawn from a large, nationally representative survey in the United States, providing comprehensive health information across diverse ethnicities, genders, and age groups. Therefore, our findings have strong external validity and can be generalized to various populations. Additionally, careful adjustment for confounding variables enhances the credibility and generalizability of the findings. The non-linear relationship between AISI and CKD was examined through smoothing curve fitting and subgroup analysis. The advantages of AISI as an inflammatory marker were demonstrated by comparing its ROC curve with those of other inflammatory markers.

This study has several limitations. First, as a cross-sectional study, our findings suggest an association between AISI and CKD. However, causal inference is not feasible due to the absence of time-series data. Furthermore, due to the limitations of the NHANES database, serum creatinine and albuminuria were measured only once for each subject, introducing potential bias in the diagnosis of CKD. Additionally, since cross-sectional data capture only a single point in time, we could not assess the long-term effects of AISI changes on CKD progression. Future longitudinal studies are required to validate these findings. Second, the sample consisted solely of US adults, limiting the applicability of these findings to children or populations from other regions, particularly those in Asia. Although we adjusted for multiple confounding factors, unmeasured ones remain, including classic inflammatory indicators such as IL-6 and TNF-α. Due to limitations in the NHANES database, we cannot obtain these inflammatory indicators, limiting our ability to fully assess the impact of other inflammatory markers on the relationship between AISI and CKD. Finally, the NHANES database’s design and specific exclusion criteria may introduce selection bias.

A promising direction for future research is the use of longitudinal data to explore the relationship between AISI and various potential causes of chronic kidney disease, such as diabetic nephropathy and hypertensive nephropathy. Additionally, the strong correlation between AISI and CKD, along with its superiority over other traditional inflammatory markers, makes it a potential tool for identifying high-risk groups for CKD. Future research could combine AISI with other indicators to develop a comprehensive predictive model, which may enhance early detection. In addition, studies of different populations, including populations outside the United States or studies of childhood cohorts, would be valuable.

## Conclusion

Our study offers new insights into the relationship between CKD and AISI. In general, AISI is positively correlated with CKD. The advantages of AISI include its low cost, ease of collection, and simple calculation, making it potentially valuable for clinical applications. However, further prospective clinical trials are required to validate the potential role of AISI in kidney disease.

## Data Availability

Publicly available datasets were analyzed in this study. This data can be found here: https://wwwn.cdc.gov/nchs/nhanes/default.aspx.

## Footnotes

1
www.empowerstats.com
