# Supplementary material for: Association between the aggregate index of systemic inflammation and CKD: evidence from NHANES 1999–2018
Source: Front Med (Lausanne). 2025 Mar 10;12:1506575. doi: 10.3389/fmed.2025.1506575 (PMC11931135; doi:10.3389/fmed.2025.1506575)
Supplement: Supplementary file 1 [file Table_1.docx]

Supplementary Table 1 Multivariate regression analysis of Ln-AISI with CKD and low eGFR

|  | **Model 1**  **OR 95% CI** | |  | | **Model 2**  **OR 95% CI** |  | **Model 3**  **OR 95% CI** |
| --- | --- | --- | --- | --- | --- | --- | --- |
| **Ln-AISI VS CKD** | | 1.39 (1.35, 1.44) |  | 1.37 (1.33, 1.42) | |  | 1.21 (1.17, 1.26) |
| Stratified by Ln-AISI quartiles | |  |  |  | |  |  |
| T1 | | ref |  | ref | |  | ref |
| T2 | | 1.18 (1.12, 1.25) |  | 1.20 (1.13, 1.27) | |  | 1.12 (1.05, 1.19) |
| T3 | | 1.63 (1.54, 1.72) |  | 1.60 (1.51, 1.70) | |  | 1.32 (1.24, 1.41) |
| *P* for trend  **Ln-AISI VS low eGFR**  Stratified by Ln-AISI quartiles  T1  T2  T3  *P* for trend | | <0.001  1.58 (1.51, 1.65)  Ref  1.29 (1.19, 1.40)  2.00 (1.85, 2.16)  <0.001 |  | <0.001  1.43 (1.36, 1.50)  Ref  1.23 (1.12, 1.35)  1.75 (1.60, 1.91)  <0.001 | |  | <0.001  1.21 (1.14, 1.28)  Ref  1.13 (1.02, 1.24)  1.38 (1.25, 1.52)  <0.001 |

OR: odds ratio

95% CI: 95% confidence interval

Model 1: no covariates were adjusted

Model 2: adjusted for gender, age, and race

Model 3: gender, age, race, Alb, BMI, education, marital status, CRP, PIR, UA, TG, LDL, diabetes, drink, hypertension, vigorous activity, moderate activity, smoke, ALT, AST.

|  |  |  |  |  |  |
| --- | --- | --- | --- | --- | --- |
