# Supplementary material for: Association between the aggregate index of systemic inflammation and CKD: evidence from NHANES 1999–2018
Source: Front Med (Lausanne). 2025 Mar 10;12:1506575. doi: 10.3389/fmed.2025.1506575 (PMC11931135; doi:10.3389/fmed.2025.1506575)
Supplement: Supplementary file 2 [file Table_2.docx]

Supplementary Table 2 Multivariate regression analysis of Ln-AISI with CKD and low eGFR (contains 25,418 CRP data, no interpolation of CRP)

|  | **Model 1**  **OR 95% CI** | |  | | **Model 2**  **OR 95% CI** |  | **Model 3**  **OR 95% CI** |
| --- | --- | --- | --- | --- | --- | --- | --- |
| **Ln-AISI VS CKD** | | 1.45 (1.38, 1.52) |  | 1.43 (1.36, 1.51) | |  | 1.28 (1.21, 1.36) |
| Stratified by Ln-AISI quartiles | |  |  |  | |  |  |
| T1 | | ref |  | ref | |  | ref |
| T2 | | 1.15 (1.06, 1.25) |  | 1.18 (1.08, 1.29) | |  | 1.10 (1.00, 1.21) |
| T3 | | 1.70 (1.57, 1.84) |  | 1.69 (1.55, 1.85) | |  | 1.44 (1.31, 1.58) |
| *P* for trend  **Ln-AISI VS low eGFR**  Stratified by Ln-AISI quartiles  T1  T2  T3  *P* for trend | | <0.001  1.55 (1.45, 1.65)  Ref  1.25 (1.11, 1.40)  1.90 (1.70, 2.12)  <0.001 |  | <0.001  1.40 (1.30, 1.51)  Ref  1.21 (1.06, 1.38)  1.67 (1.48, 1.89)  <0.001 | |  | <0.001  1.19 (1.09, 1.29)  Ref  1.08 (0.94, 1.24)  1.33 (1.16, 1.53)  <0.001 |

OR: odds ratio

95% CI: 95% confidence interval

Model 1: no covariates were adjusted

Model 2: adjusted for gender, age, and race

Model 3: gender, age, race, Alb, BMI, education, marital status, PIR, UA, TG, LDL, diabetes, drink, hypertension, vigorous activity, moderate activity, smoke, ALT, AST.

|  |  |  |  |  |  |
| --- | --- | --- | --- | --- | --- |
