# Supplementary material for: Association between the aggregate index of systemic inflammation and CKD: evidence from NHANES 1999–2018
Source: Front Med (Lausanne). 2025 Mar 10;12:1506575. doi: 10.3389/fmed.2025.1506575 (PMC11931135; doi:10.3389/fmed.2025.1506575)
Supplement: Supplementary file 3 [file Table_3.docx]

Supplementary Table 3: Multivariate regression analysis of Ln-AISI and CKD and low eGFR (analysis after multiple imputation of data from the entire population 1999-2018)

|  | **Model 1**  **OR 95% CI** | |  | | **Model 2**  **OR 95% CI** |  | **Model 3**  **OR 95% CI** |
| --- | --- | --- | --- | --- | --- | --- | --- |
| **Ln-AISI VS CKD** | | 1.31 (1.27, 1.35) |  | 1.21 (1.18, 1.25) | |  | 1.14 (1.11, 1.18) |
| Stratified by Ln-AISI quartiles | |  |  |  | |  |  |
| T1 | | ref |  | ref | |  | ref |
| T2 | | 1.27 (1.20, 1.33) |  | 1.16 (1.10, 1.22) | |  | 1.09 (1.03, 1.15) |
| T3 | | 1.01 (0.97, 1.05) |  | 0.94 (0.90, 0.99) | |  | 0.95 (0.91, 1.00) |
| *P* for trend  **Ln-AISI VS low eGFR**  Stratified by Ln-AISI quartiles  T1  T2  T3  *P* for trend | | 0.3267  1.69 (1.61, 1.77)  Ref  1.52 (1.39, 1.66)  1.36 (1.27, 1.46)  <0.001 |  | 0.0257  1.39 (1.32, 1.46)  Ref  1.22 (1.11, 1.35)  1.15 (1.06, 1.25)  0.0006 | |  | 0.0900  1.18 (1.11, 1.25)  Ref  1.12 (1.01, 1.24)  1.04 (0.95, 1.14)  0.3542 |

OR: odds ratio

95% CI: 95% confidence interval

Model 1: no covariates were adjusted

Model 2: adjusted for gender, age, and race

Model 3: gender, age, race, Alb, BMI, education, marital status, PIR, UA, TG, LDL, diabetes, drink, hypertension, vigorous activity, moderate activity, smoke, ALT, AST.

|  |  |  |  |  |  |
| --- | --- | --- | --- | --- | --- |
