# Supplementary material for: Association between the aggregate index of systemic inflammation and CKD: evidence from NHANES 1999–2018
Source: Front Med (Lausanne). 2025 Mar 10;12:1506575. doi: 10.3389/fmed.2025.1506575 (PMC11931135; doi:10.3389/fmed.2025.1506575)
Supplement: Supplementary file 4 [file Data_Sheet_1.docx]

**Threshold and saturation effect analysis explained:**

First, we applied smooth curve fitting to examine whether the independent variable, Ln-AISI, could be partitioned into intervals. Then, we used segmented regression (also known as piecewise regression), which fits separate line segments to each interval. To determine if a threshold exists, we performed a log-likelihood ratio test, comparing the non-segmented model (single line) to the segmented regression model.

Next, we identified the inflection point (the point where the segments connect) by maximizing the model’s likelihood using a two-step recursive method:

Step 1:
We narrowed down the potential inflection point to a 10th percentile range of the independent variable. Specifically, we tested 19 segmented regression models using percentile points from 5% to 95%, incremented by 5%. For each of the 19 models, we tested different percentile points as potential inflection points and selected the one with the highest likelihood. We then refined the range of the inflection point to within +/- 4% of the percentile point that yielded the highest likelihood, which we referred to as Kmin and Kmax.

Step 2:
To determine the precise inflection point, we performed a recursive method within the narrowed range of Kmin and Kmax. In this step, we ran three models using inflection points at the 25% (Q1), 50% (Q2), and 75% (Q3) percentiles of the narrowed range. We selected the quartile point that provided the highest likelihood and further reduced the range of Kmin and Kmax to +/- 25% of that quartile point. This recursive process continued until we identified the precise inflection point that gave the highest likelihood for the segmented regression model.

**sample size calculation:**

Main statistical indicators:

Ratio of ratios (OR) = 1.24

Number of covariates: 20

Statistical efficacy: 90%

Significance level: 0.05

Prevalence of CKD: 20.31%

We used a sample size formula commonly used in cross-sectional studies to estimate sample: based on the ratio of ratios (OR) and prevalence of CKD: n=(1−p)(Zα/2+Zβ)2×p×(1−p)×(ln(OR))2

Twenty covariates were included in our study. Although covariates affect the required sample size in sample size calculations, this does not only depend on the number of covariates, but is also influenced by the effect size of each covariate and the correlation between covariates. Thus, the effect of covariates on sample size is complex and does not increase exactly linearly. We used a rule of thumb assuming that each covariate requires a minimum of 10 samples, so preliminary estimates adjusted the sample size to:

nadjusted=47×20=940​

he total number of participants in our study, 50768, is much larger than the minimum sample size calculation required, ensuring the scientific validity and reliability of our study.
