# Supplementary material for: Association between the aggregate index of systemic inflammation and CKD: evidence from NHANES 1999–2018
Source: Front Med (Lausanne). 2025 Mar 10;12:1506575. doi: 10.3389/fmed.2025.1506575 (PMC11931135; doi:10.3389/fmed.2025.1506575)
Supplement: Supplementary file 5 [file Image_1.pdf]

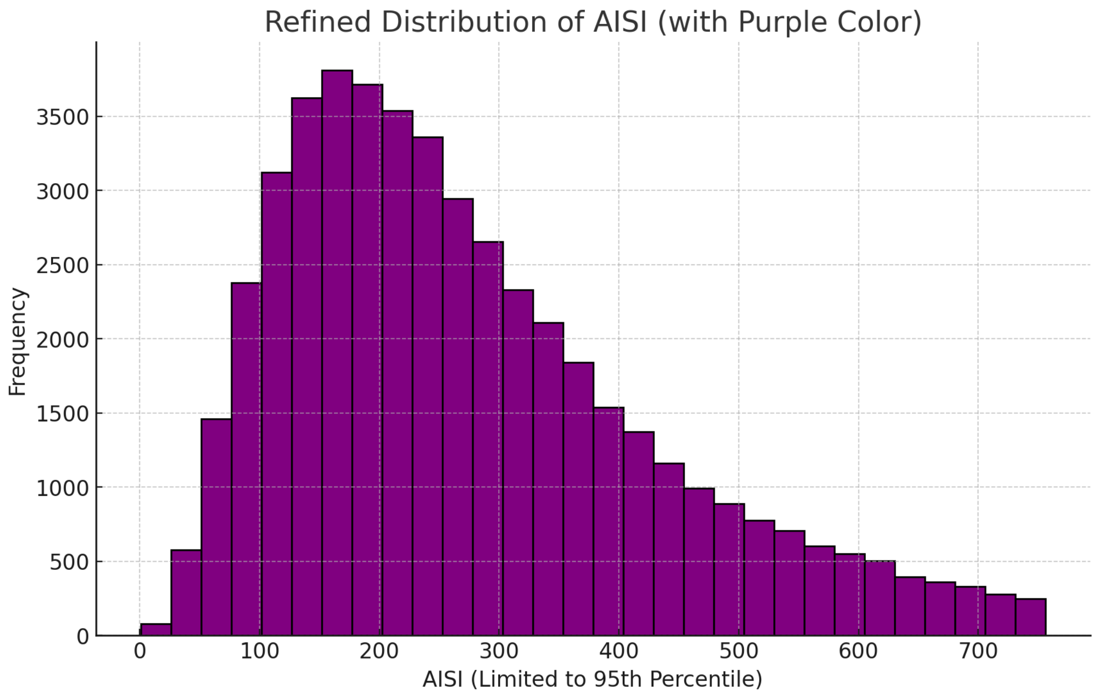

Supplementary Figure 1: Refined Distribution of AISI

This histogram shows the distribution of the aggregate index of systemic inflammation (AISI) limited to the 95th percentile. The x-axis represents AISI values, while the y-axis represents the frequency of occurrences within the dataset. The purple bars indicate the frequency distribution across different AISI values, with the highest density observed between 100 and 300.
