# Supplementary material for: Association between the aggregate index of systemic inflammation and CKD: evidence from NHANES 1999–2018
Source: Front Med (Lausanne). 2025 Mar 10;12:1506575. doi: 10.3389/fmed.2025.1506575 (PMC11931135; doi:10.3389/fmed.2025.1506575)
Supplement: Supplementary file 6 [file Image_2.pdf]

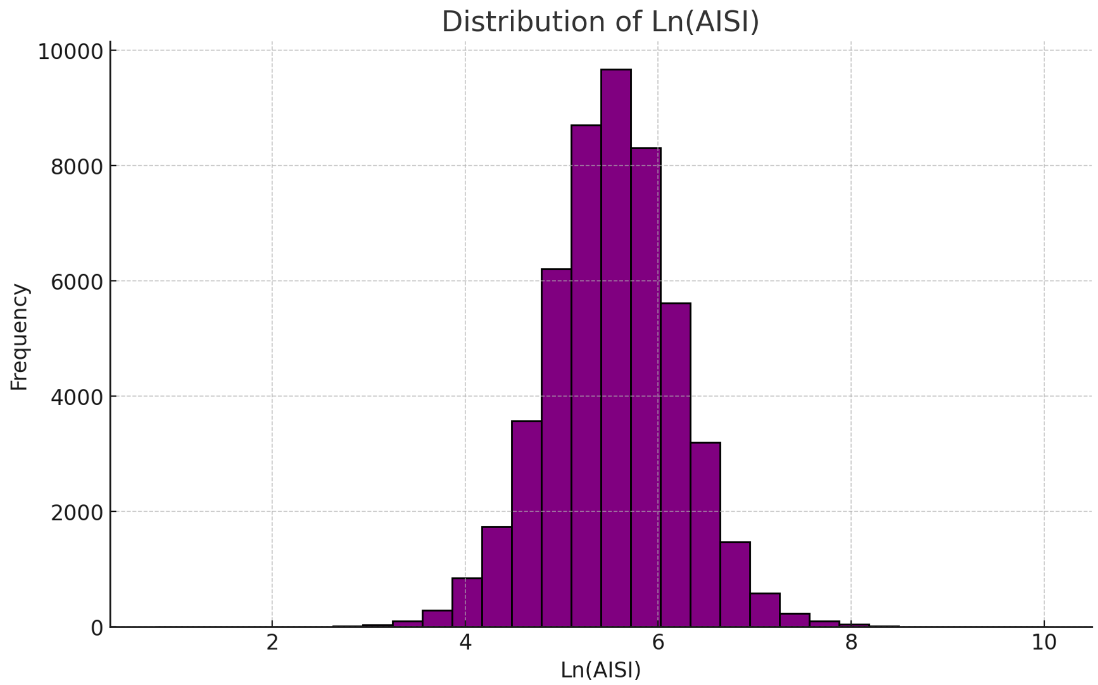

Supplementary Figure 2: Distribution of Ln(AISI)

This histogram represents the logarithmic transformation of the aggregate index of systemic inflammation (Ln(AISI)) for the study population. The x-axis shows the natural logarithm of AISI values, while the y-axis indicates the frequency of occurrences for each value range. The distribution appears approximately normal, with the highest frequency centered around an Ln(AISI) value of 6.
