# Supplementary figures and images for: Association between the aggregate index of systemic inflammation and CKD: evidence from NHANES 1999–2018
Source: Front Med (Lausanne). 2025 Mar 10;12:1506575. doi: 10.3389/fmed.2025.1506575 (PMC11931135; doi:10.3389/fmed.2025.1506575)

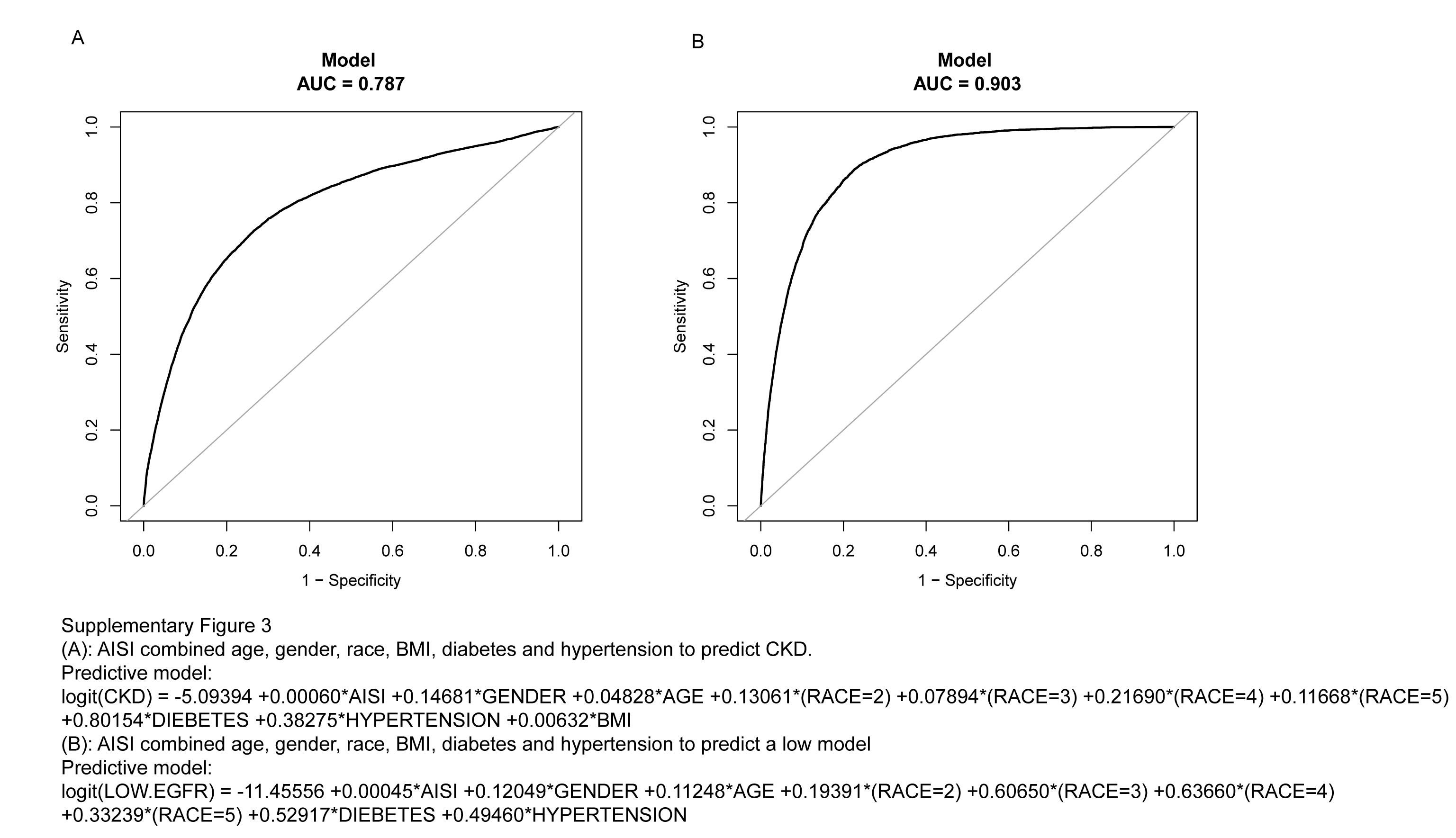

Supplement: Supplementary file 7 [file Image_3.tif]
